# Sustainable and Eco-Friendly Single- and Multilayer Polyester Foils (Laminates) from Polylactide and Poly(Ethylene 2,5-Furandicarboxylate)

**DOI:** 10.3390/molecules30010178

**Published:** 2025-01-04

**Authors:** Sandra Paszkiewicz, Izabela Irska, Konrad Walkowiak, Filip Włodarczyk, Magdalena Zdanowicz, Elżbieta Piesowicz, Mateusz Barczewski

**Affiliations:** 1 Department of Materials Technologies, Faculty of Mechanical Engineering and Mechatronics, West Pomeranian University of Technology in Szczecin, Piastow Av. 19, 70-310 Szczecin, Poland; izabela.irska@zut.edu.pl (I.I.); wk42388@zut.edu.pl (K.W.);; 2 Center of Bioimmobilisation and Innovative Packaging Materials, Faculty of Food Sciences and Fisheries, West Pomeranian University of Technology in Szczecin, Janickiego 35, 71-270 Szczecin, Poland; 3 Polymer Processing Division, Institute of Materials Technology, Faculty of Mechanical Engineering, Poznan University of Technology, Piotrowo 3, 61-138 Poznan, Poland; mateusz.barczewski@put.poznan.pl

**Keywords:** barrier properties, polylactide, PLA, poly(ethylene 2,5-furanodicarboxylate), PEF, packaging, biobased polyesters, laminates

## Abstract

Packaging materials mainly serve the function of protecting products. The most common representative of this group is poly(ethylene terephthalate) (PET), which is not biodegradable and therefore, its waste might be burdensome to the environment. Thus, this work aims to develop outlines for obtaining polyester-based systems, preferably biobased ones, intended for the packaging industry and their detailed characterization. The obtained multilayer systems based on two biobased thermoplastic polyesters, i.e., poly(ethylene 2,5-furandicarboxylate) (PEF) and the “double green” polylactide (PLA), were subjected to various analyses, i.e., UV-Vis spectrophotometry, microscopic evaluation, tensile tests, differential scanning calorimetry (DSC), oxygen transmission rate (OTR), water absorption tests, surface analyses, and biofilm formation. The best possible arrangement was selected in terms of the packaging industry. It was proven that the elastic modulus was significantly higher for multilayer systems, whilst higher-strength parameters were observed for PLA single foils. Regardless of thickness, PLA and PEF foils exhibit similar absorption values in cold water. Moreover, PEF foils demonstrated significantly better barrier properties towards oxygen gas compared to PLA foils of the same thickness. However, a multilayer system composed of two PLA foils with a single inner PEF foil had an OTR value only slightly higher than that of the PEF foil alone. PEF was also found to be a material that exhibited a limited formation of bacterial biofilm, particularly strains of *S. aureus* and *E. coli*. All of the above findings clearly confirm the sensibility of the research topic undertaken in this work on the application of biobased thermoplastic polyesters in the packaging industry.

## 1. Introduction

The worldwide lifestyle and dietary pattern changes spurred by industrialization, urbanization, economic progress, and market globalization have prompted significant alterations [1,2]. Nowadays, the majority of food consumed is packaged not only for containment but also to ensure its protection throughout the entire production and distribution process, from manufacturing to consumption. Consequently, there is a growing demand for safe, minimally processed, and “fresh” food items. This demand has necessitated the food industry to innovate in used materials, with the primary challenge being the development of novel packaging systems that can uphold both the safety and quality of packaged foods [3].

In this manner, food packaging serves crucial roles in preserving food products by preventing physical, chemical, and/or microbiological contamination, thereby averting the deterioration of food quality. Moreover, it offers essential information to consumers, including nutritional details, recommended consumption dates, and instructions for proper food storage [3,4,5]. Additionally, packaging often provides details about its material composition, recyclability, and disposal instructions after its useful life. Plastics are particularly favored for food packaging due to practical and economic advantages such as its affordability, lightweight nature, ease of processing, and suitability for integrated production lines. They also exhibit greater resilience compared to alternative materials like ceramics, glass, or cardboard [6,7,8].

Indeed, the packaging industry accounts for a significant share of polymer production demand. Plastic packaging materials, mainly polymers, typically contain additives such as plasticizers, antioxidants, pigments, and fillers to ensure desired functionality [9]. With global plastic production surpassing 250 million tons annually in recent years, coupled with the prevalent short-term use of packaging products, they constitute a major source of plastic waste [10]. Consequently, waste management strategies prioritize material and energy recovery methods. While some plastic waste can be recycled, most packaging residues, especially those from the food packaging sector, end up in landfills yearly due to technical and economic constraints [11,12]. This results in a substantial accumulation of plastic waste without proper energy or material recovery.

Currently, plastics are predominantly derived from petrochemicals, which originate from fossil oil and gas. Approximately 4% of global oil and gas output, non-renewable resources, serve as raw materials for plastics, with an additional 3–4% utilized for energy in their manufacturing process [13]. Consequently, employing non-renewable and non-biodegradable polymers for food packaging raises environmental concerns due to the depletion of non-renewable petrochemical sources during production and the accumulation of substantial plastic waste post-use. These issues have prompted escalating interest in adopting more sustainable polymers for food packaging, namely biopolymers, encompassing biobased and biodegradable variants [12]. This shift aligns with the worldwide movement towards shifting to a more sustainable economy, thereby fostering the expansion of global bioplastic production. In this context, bioplastics—plastics derived from renewable sources and/or capable of biodegradation—emerge as a potential solution to address these pressing concerns [13]. Utilizing bioplastics helps diminish reliance on fossil fuels, mitigates greenhouse gas (GHG) emissions, fosters renewable energy generation, and enhances resource efficiency. Furthermore, a portion of the current bioplastic production volume is recycled in conjunction with traditional plastics, such as biobased polyethylene (PE) in the PE stream or biobased poly(ethylene terephthalate) (PET) in the PET stream, thereby contributing to a more effective waste management system.

One of the most commonly used polymers for packaging, derived from renewable sources and produced via fermentation to obtain its monomer, is polylactide (PLA). The utilization of biobased PLA aims to mitigate environmental issues associated with the extensive use of synthetic polymers derived from non-renewable sources. PLA stands out as a well-established biopolymer with mechanical properties that rival those of petroleum-based polymers. The production process of PLA offers several advantages over other biopolymer manufacturing methods: (a) the lactide monomer is derived from lactic acid, sourced from the fermentation of renewable materials like maize, sugarcane, potato, and corn; (b) it conserves energy; (c) it reduces landfill volume; (d) it enables the creation of compostable hybrid paper–plastic packaging; (e) it holds significant potential for enhancing physical properties through material modifications [14]. With an annual production rate of approximately 140,000 tons, PLA is the most extensively utilized biopolymer in the food packaging sector. Using PLA as a food packaging material, including bottles, trays, clamshell containers, boxes, and films, offers various environmental advantages supporting its adoption. These benefits stem primarily from the utilization of renewable resources and result in favorable outcomes across multiple impact categories, including a reduction in global warming impact when compared to PET alternatives. The environmental advantages of using PLA for food packaging (such as bottles, trays, clamshell containers, boxes, and films) [15,16,17,18,19,20,21,22] support its implementation. These benefits primarily stem from using renewable resources, such as carbon uptake [16,23], and are evident across various impact categories, including reduced global warming impact compared to PET alternatives [15,16,19,20].

Due to its necessity and extensive packaging utilization in the food industry, the demand for PLA is expected to increase by 30% over the next two years. PLA has received a Generally Recognized as Safe (GRAS) classification from the United States Food and Drug Administration (FDA) and is deemed safe for all food packaging applications [24]. With its high strength and stiffness, PLA is viewed as a promising alternative to petroleum-based materials [25]. However, its inherent brittleness has limited its potential applications. Moreover, PLA exhibits only moderate barrier properties against gasses, vapors, and organic compounds, which may restrict its suitability as a packaging material. Therefore, understanding the mass transport properties of PLA and addressing these transport challenges is crucial. To enhance its barrier properties, significant advancements can be made by integrating nano-additives such as two-dimensional clay nanofillers, which create tortuosity in diffusing molecules’ paths, thereby increasing the effective diffusion length [26]. Alternatively, combining PLA with materials possessing superior properties can also lead to improvements [27,28].

Among various renewable starting materials utilized for bioplastic production, furan-based monomers have garnered significant interest, with 2,5-furandicarboxylic acid (FDCA) being a prominent example. Its popularity primarily stems from its application in synthesizing poly(ethylene 2,5-furanoate) (PEF), which is currently regarded as the most viable biobased alternative to PET due to its highly favorable physical and mechanical properties. Notably, PEF demonstrates superior barrier performance and more desirable thermal and mechanical attributes compared to PET. Specifically, it boasts a higher glass transition temperature (T_g_) of 85 °C versus 76 °C, a lower melting temperature (T_m_) of 211 °C compared to 247 °C [29], a 1.6 times higher Young’s modulus [29], an 11 times lower oxygen permeability [30], a 19 times lower carbon dioxide permeability [31], and a 5 times lower water diffusion coefficient [32]. Furthermore, the production of PEF is estimated to reduce non-renewable energy consumption by approximately 40–50% and greenhouse gas emissions by approximately 45–55% compared to PET [33]. PEF has been effectively employed in manufacturing thermoformed packaging containers, bottles, films, and fibers [22]. However, its discoloration remains a significant practical challenge that must be addressed to expand the scope of its applications, particularly in packaging contexts. For instance, a multilayer recyclable PET container incorporating semicrystalline PEF as an internal barrier layer [34] has been fabricated using injection molding, either in a single step or through over-molding techniques. The utilization of semicrystalline PEF enables a reduction in the thickness of the barrier layer in thermoformed containers, consequently reducing the overall weight of an all-PET bottle. This weight reduction can reach up to 5% for every weight percent of PEF added while simultaneously enhancing the shelf life of the container [34]. Additionally, PEF/PET blend bottles can be manufactured via injection molding to produce the preform, followed by stretch blow molding to form the bottle, under conditions similar to those used for PET [35]. Moreover, environmentally friendly melt compounded PET-G/PEF blends with varied component weight ratios based on post-consumer foils of PET-G and PEF have been prepared and after considering the synergistic effect of improving thermal properties for the PET-G/PEF 80/20, the water diffusion for the PET-G/PEF 50/50, with the optimum characteristic phase transition temperatures and mechanical parameters for the blends 80/20 and 70/30, and above all the market price of dimethyl furan-2,5-carboxylate of about USD 800/kg [36], the most promising system for the packaging and other industries was found to be the one system containing 80 wt.% of PET-G and 20 wt.% of PEF [37].

The replacement of PET with furan-based counterparts extends beyond PEF despite its prominent status [22]. The broader category of furan-based polyesters (referred to as FBP) includes polymers such as poly(trimethylene-2,5-furandicarbocylate) (PTF) or poly(pentamethylene-2,5-furandicarboxylate) (PPeF), which offer a wide range of customizable thermal and mechanical properties depending on the number of methylene groups in the glycol moiety [38]. This versatility enables their application in various PET-like roles, including rigid or flexible packaging and fibers. PTF, in particular, has garnered industrial interest from major players like DuPont and ADM due to its exceptional barrier properties, surpassing even those of PEF, especially against CO_2_. Additionally, PPeF, despite being an amorphous polymer, shows considerable promise. In its rubbery state, it can be easily processed into freestanding flexible films characterized by remarkable thermal stability and mechanical resilience typical of elastomeric materials, with immediate shape recovery after deformation [39]. Furthermore, PPeF exhibits exceptional barrier properties against O_2_ and CO_2_, comparable to an ethylene-vinyl alcohol copolymer with 32% ethylene content [40].

Multilayer packaging is an innovative approach that combines the distinct characteristics of various polymers to create a package with enhanced performance in terms of improved protection and durability [41,42]. In many instances, a single polymer layer cannot fulfill all the requirements of food packaging, which include containments (such as strength and sealability), protection/preservation (moisture, gas, light, flavor/odor barrier), machinability (tensile strength, softening, slip, rigidity, pliability, and heat resistance), promotion, and convenience, all while ensuring cost-effectiveness and adhering to food safety standards [41]. Therefore, the objective behind the development of multilayer packaging is to create a single packaging structure that possesses multiple functional properties to meet the complex functional demands of packaging.

Typically, existing multilayer packages comprise 3 to 12 layers, whereas in food packaging specifically, they usually consist of 3 to 7 layers [43]. Synthetic polymer-based packaging materials have gained extensive use in the food industry over the years compared to conventional materials like metal, glass, and paper [44]. This preference is due to their affordability, versatility, functionality, and scalability. Multilayer polymeric packages provide added benefits, such as combining functional and barrier properties of the individual polymers and reducing the overall material usage. This results in the production of thinner, lighter, and more compact packages [43,45].

Polyesters, primarily PET, are commonly employed as one of the layers in multilayer packaging films because of their superior mechanical strength, barrier properties, and transparency. However, as mentioned above, PEF and PLA can successfully replace PET in these applications. Examples of industrial use of biopolyesters include the solution of Mitsubishi Plastics, Inc. (Tokyo, Japan), which started sales of its new product “DIAMIRONTM MF P-TYPE”, which is a co-extruded multilayer film used for food packagings such as hams and sausages as well as for other various uses [46]. Another example is a Q-Top BioPeel, (Quantum Packaging, Musselburgh, Scotland), a lidding film entirely made out of PLA that exhibits a good gas and aroma barrier, mechanical resistance against puncturing and tearing, and excellent green credentials [47].

The inevitable need for the development of packaging products with superior barrier properties for the food industry necessitates intensive research in the field of both material and technological solutions. This research constitutes preliminary work on developing technology for manufacturing barrier films based on biobased polyesters (PLA and PEF). As part of these studies, combining multilayer systems (laminates) with a highly barrier-resistant PEF layer coated with PLA with a high concentration of a D-enantiomer, i.e., in a polymer system with an amorphous structure, was verified. The research framework aimed at creating base knowledge for further technological-oriented studies, recognizing and mitigating material–structural effects that may pose a problem in the process of combining layers. It was verified how the thickness of the foil in conjugated and mono-polymer systems influenced the mechanical and thermal properties, structure, barrier, and surface properties of PLA, PEF, and PLA-PEF-PLA laminates.

## 2. Results and Discussion

### 2.1. UV-Vis Spectrophotometry and Microscopic Evaluation of Polyesters’ Foils

**Figure 1:**
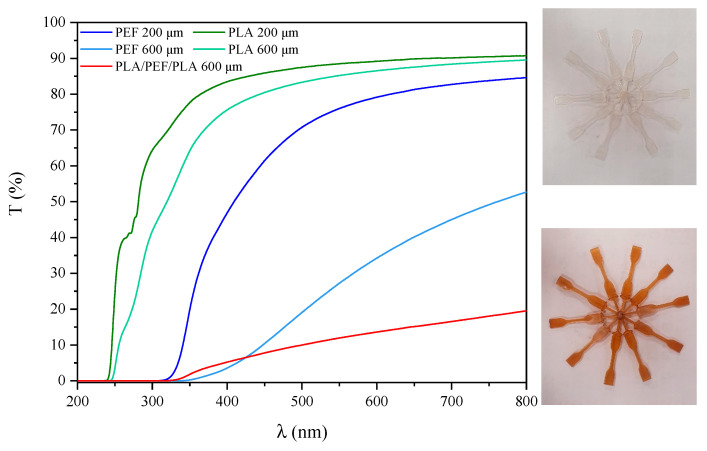
UV-Vis transmittance spectra and the photos of the injected dumbbell-shape samples: PLA (upper), PEF (down).

**Figure 2:**
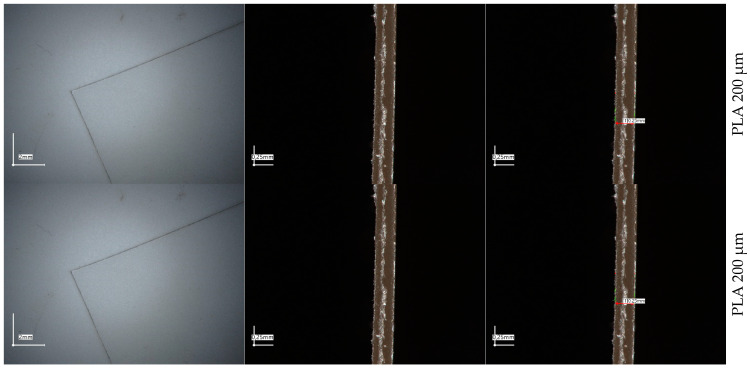

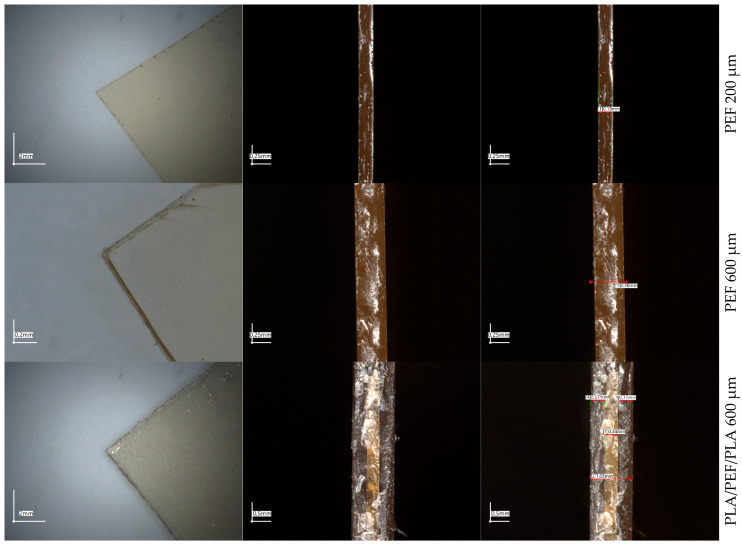
A compilation of micrographs of polyester foils with a transverse view from a 4K digital microscope.

In Figure 1, the UV-Vis transmittance spectra for PLA 200 μm, PLA 600 μm, PEF 200 μm, PEF 600 μm, and PLA/PEF/PLA 600 μm multilayer systems are shown. As expected, considering the color of the analyzed materials, PEF revealed lower transmitances in the whole wavelength range than PLA: PLA foils were colorless, while PEF foils were orange/brownish. Despite using a thermal stabilizer during the synthesis (Irganox 1010), a slight process-induced discoloration of the material occurred. Nevertheless, as the material exhibited good mechanical properties, LVN of approximately 0.5, and values of phase transition temperatures (especially T_g_) (discussed later in the article) comparable to literature data [48,49], the color change in the PEF film is probably not associated with the degradation. The influence of foil thickness on transparency was also demonstrated (Figure 2). Films with a thickness of 600 μm showed lower transparency than their counterparts with a thickness of 200 μm. The three-layer foil showed by far the least transparency among all those tested. Furthermore, it is particularly interesting to note that PLA and PEF demonstrate distinct behavior in the ultraviolet region, especially within the UV-C (100 to 280 nm) and UV-B (280 to 315 nm) ranges. The former transmitted a significant portion of light, whereas the latter effectively blocked UV light within the specified range (<315 nm). In both the short-wavelength UV-A and medium-wavelength UV-B ranges of the transmission spectra, the multilayer PLA/PEF/PLA system exhibits a resemblance to that of PEF. In both samples, energy in this region is absorbed by aromatic sequences in the polymer structure or, more specifically, by groups of atoms with delocalized electrons (furan rings conjugated with carbonyl groups) [50]. Strong UV-shielding properties in the UV region can be considered beneficial from the point of view of packaging applications. It can prevent packed food from a loss of nutritional value and alteration of organoleptic properties, e.g., fat photo-oxidation, vitamin degradation, and fresh food discoloration while increasing the product shelf life [50,51].

Polyester foils and laminates were subjected to microscopic examination. Based on the foil thickness measurement, it was shown that the foils have slightly different thicknesses than expected; i.e., the PLA200 foil is approximately 250 μm thick according to microscopic measurements, while the PLA600 foil is approximately 480 μm thick. These are approximate measurements made in one place of the foil. Nonetheless, for tensile and OTR tests, the obtained foils were measured at several points to confirm the assumed thickness. Particularly noteworthy is the microgram presented in the figure below of the three-layer PLA/PEF/PLA foil, where the individual layers of the foil can be clearly distinguished. This confirms that the three-layer system was prepared correctly; there is no melting of individual foils constituting the system.

Additionally, micrographs of the foils at low magnification are presented in the left panel to compare their color. PLA foils were transparent, while PEF foils were rather slightly yellowish. The PLA/PEF/PLA laminate is also not transparent due to the use of an internal PEF layer. Digital micrographs confirm the observations made using a UV-Vis spectrophotometer.

### 2.2. DSC Analysis

**Figure 3:**
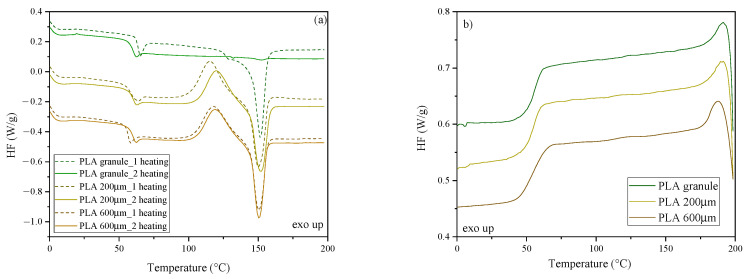
DSC thermograms recorded during the first and second heating (**a**) and cooling (**b**) for PLA.

**Figure 4:**
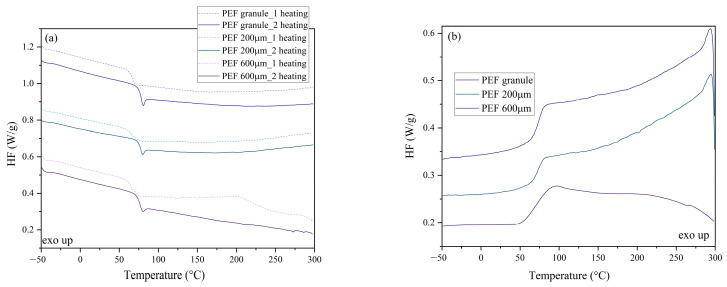
DSC thermograms recorded during the first and second heating (**a**) and cooling (**b**) for PEF.

**Figure 5:**
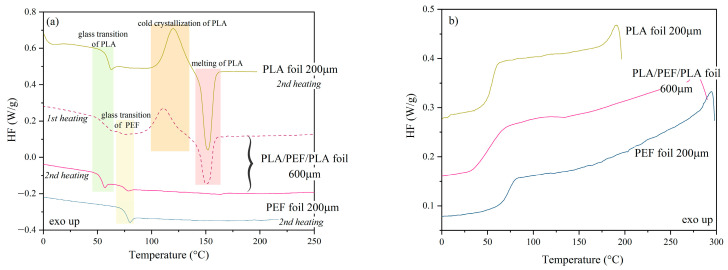
DSC thermograms recorded during the first and second heating for the multilayer system of PLA/PEF/PLA and second heating of PLA at 200 μm and PEF at 200 μm (**a**), and for all previously mentioned during cooling (**b**).

The characteristic phase transformation temperatures of the analyzed polyesters were determined using differential scanning calorimetry (DSC). Figure 3, Figure 4 and Figure 5 show DSC thermograms recorded during the first and second heating and cooling. Additionally, Table S1 (Supplementary Materials) lists the values corresponding to thermal effects, i.e., glass transition temperature (T_g_)_,_ changes in specific heat (ΔC_p_), cold crystallization temperature (T_cc_), and the corresponding change in cold crystallization enthalpy (ΔH_cc_), the melting temperature, and the corresponding change in the enthalpy of melting (ΔH_m_). Moreover, Table S1 lists the determined crystallinity values (X_c_).

Figure 3a shows DSC thermograms recorded during the first heating (dashed line) and the second heating for PLA in the form of granules and foils with 200 μm and 600 μm thicknesses. The influence of subsequent processing cycles, i.e., injection and compression molding, was observed on the values of characteristic phase transition temperatures. From the thermograms recorded during the first heating, one can see that the highest glass transition temperature exhibited the “unprocessed” PLA material, i.e., for which the measurement was made for a granule (while the T_g_ values for PLA200 and PLA600 films are similar to one another: a difference of 1.5 °C (Table S1)). The increased T_g_ observed in the case of untreated PLA is undoubtedly related to its increased X_c_ compared to PLA processed and rapidly cooled from the melt (~40% for polymer pellets vs. ~2% for polymer foils). The increased amount of the crystalline phase restricts the movement of polymer chains in the amorphous phase, shifting T_g_ towards higher temperatures. The noticeable impact of additional processing in the molten state, the ability to form crystalline domains in the cold crystallization process, may indicate the occurrence of thermo-oxidative and thermomechanical-originated degradation phenomena accompanying the processing [52,53]. Despite the amorphous nature of the PLA grade used, resulting from the significant D-enantiomer content, shortening the length of the main chains of the PLA macromolecules leads to their increased mobility at elevated temperatures. This effect translates into an ability to reorganize the structure in the solid-state crystallization process. The thermograms recorded during the second heating show that the T_g_ values between the granulate and the foils are less pronounced. It can also be observed that PLA (granule) melts at 151.8 °C during the first heating, while during the second heating, the melting peak at 152.6 °C has a very low melting enthalpy (ΔH_m_) corresponding to a degree of crystallinity of 0.9%. In turn, the thermograms for PLA foil show both endothermic (melting) and exothermic transformations related to the cold crystallization process. In the case of foils, the values of melting temperatures (T_m_) and cold crystallization (T_cc_) are comparable (Table S1), regardless of the foil thickness; only in the case of PLA at 200 μm, a slight shift in the cold crystallization peak towards higher temperatures is observed during the second heating. These differences probably result from the tested sample’s different thermal inertia, which is an effect of its thickness, not from changes in material structure.

Figure 3b shows DSC thermograms recorded during cooling of PLA and PLA 200 μm and PLA 600 μm films. The glass transition temperature values were similar in all cases (Table S1). Based on the DSC analysis performed for PLA, it can be concluded that this polyester does not crystallize from the melt during cooling, but for both PLA foils, the crystalline phase is formed by the solid-state reorganization of the macromolecules. These results suggest that processed PLA crystallization is considerably faster than virgin polyester. Pantani and co-workers [54] confirmed these findings from the investigation of injection-molded and extruded PLA. According to the authors, both processing methods have a significant impact on accelerating PLA crystallization.

Figure 4a shows DSC thermograms for PEF recorded during the first (dashed line) and second heating (solid line), and 4b shows DSC thermograms recorded during cooling from the melt. It can be concluded that under the measurement conditions, PEF was an amorphous polyester—no endothermic or exothermic peaks were observed at a heating/cooling rate of 10 °C/min. The glass transition temperature values during the first and second heating and cooling for PEF and PEF foil are similar (Table S1). Nonetheless, it was observed that T_g_ values are higher during the second heating, which results from changes in the structure of the material resulting from the long-range chain arrangements subjected to heating and cooling at a constant rate of 10 °C/min [55].

Figure 5 shows DSC thermograms recorded during the first heating (dashed line) and second heating (solid line) (Figure 5a) and cooling (Figure 5b) for the PLA/PEF/PLA multilayer foil, compared to thermograms for PLA 200 μm and PEF 200 μm foils. The individual phase transitions are additionally highlighted in color to make it easier to follow the individual transformations in the multilayer system resulting from the structure. From the thermogram of the first heating for PLA/PEF/PLA foil, the phase transitions associated with PLA glass transition, PEF glass transition, and cold crystallization, and PLA melting, are visible. In contrast, the thermogram of the second heating shows only glass transition for both polyesters.

Based on the analysis of thermograms recorded during cooling (Figure 5b), it can be concluded that the multilayer system does not crystallize from the melt—only the glass transition (materials transformed into a glassy state below the T_g_) is visible for the analyzed materials. In this case, one T_g_ value was determined for the PLA/PEF/PLA film, and this is the glass transition corresponding to PLA. It was not possible to determine the glass transition temperature value for PEF, as both polymers had comparable T_g_ values during cooling (Figure 5b), and the thermal effect from the glass transition of PEF unfortunately could not be determined. In this case, it would be necessary to measure with a different heating/cooling rate to separate the overlapping effects.

### 2.3. Mechanical Properties

**Figure 6:**
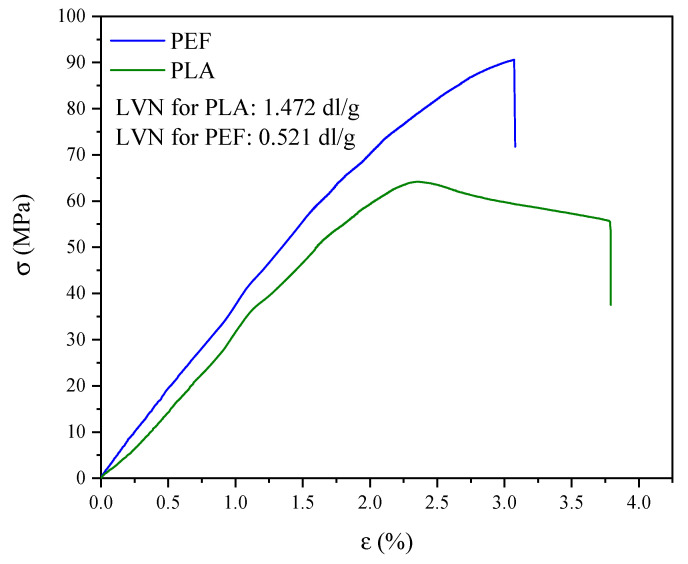
Representative stress-strain curves for PEF and PLA (dumbbell-shape samples).

**Figure 7:**
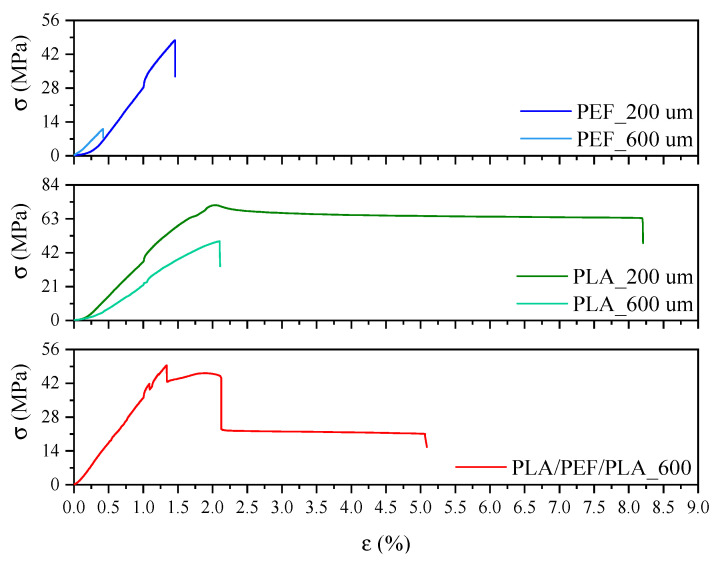
Representative stress-strain curves for PEF-based and PLA-based foils.

**Figure 8:**
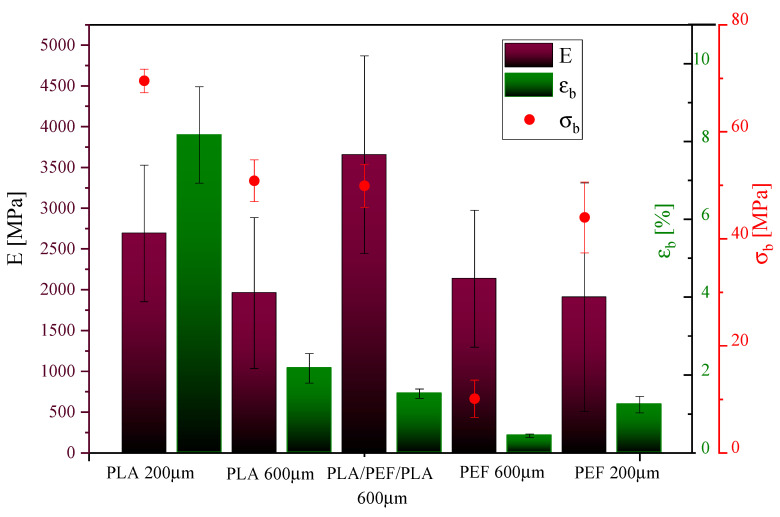
Mechanical properties of the tested polyesters, where E is the modulus of elasticity calculated from deformations 0.05% to 0.25%, σ_b_ is the stress at break, and ε_b_ is the elongation at break.

The mechanical properties of the tested polyesters were determined by uniaxial tensile testing (Figure 6 and Figure 7). Values such as the elastic modulus (E), stress at break (σ_b_), and elongation at break (ε_b_) are summarized in Figure 8. Figure 6 shows representative curves of injection molding samples of PLA and PEF, while Figure 7 shows representative curves of polyester foils. When analyzing the results of mechanical tests for the dumbbell-shaped samples (Figure 6), it can be seen that PEF turned out to be a slightly “stiffer” polyester than PLA: it showed a higher value of the elastic modulus (by about 8%), a higher value of stress at break (by about 15%), and a lower value of elongation at break (by about 40%). In turn, when comparing the polyester foils, it is clearly visible that the strength characteristics depended on the foil thickness (Figure 7 and Figure 8). A decrease in strength characteristics was observed for PLA with increasing film thickness. A similar relationship was observed for PEF foils; however, the differences were minor. On the other hand, comparing PLA and PEF foils at the same thickness, it was found that PLA at 200 μm showed a higher elastic modulus than PEF at 200 μm, but already for thicker films (600 μm), higher values were observed for PEF series. PLA foils also exhibited higher values of stress and elongation at break. In both cases, the effect of film thickness on σ_b_ and ε_b_ values was observed—a decrease with increasing foil thickness. The most promising choice, considering the value of the elastic modulus, turned out to be the PLA/PEF/PLA multilayer system, where a higher modulus value of about 1600 MPa was observed if compared to PLA at 600 μm, and 1500 MPa when compared to PEF at 600 μm. The high parameter value can be related to the good adhesion of the polyester layers in the material. In Figure 7, the superimposed layer breaks are visible for the laminates in the stress–strain curve. The “step” at ε_b_ ca. 2% is assigned to the PEF internal layer, and the last “step” at ε_b_ ca. 5.1% is the break of PLA. Due to the adhesion to other layers, restricting mobility, the value is lower than the pure PLA.

The determined LVN values are inserted in Figure 6. The LVN values can be referred to as molecular masses [56]. Both analyzed polyesters showed relatively high values of LVN, in comparison with PET used in film extrusion or injection molding, which exhibits LVN values in the range of 0.65–0.9 dl/g [57]. The LVN measurement was taken for the granule right after the synthesis process in the case of PEF, while in the case of PLA, it was an unprocessed granule. Since the PLA analyzed was a commercial material, a higher LVN value was assumed (PLA showed 2.83 times higher LVN value than PEF). Nevertheless, the value of 0.521 dl/g is comparable to previously obtained LVN values for PEF synthesized according to the same procedure [58], which confirms the reproducibility of the conditions for obtaining polyesters by polycondensation in the molten state.

### 2.4. Water Sorption and Oxygen Transmission Rate

**Figure 9:**
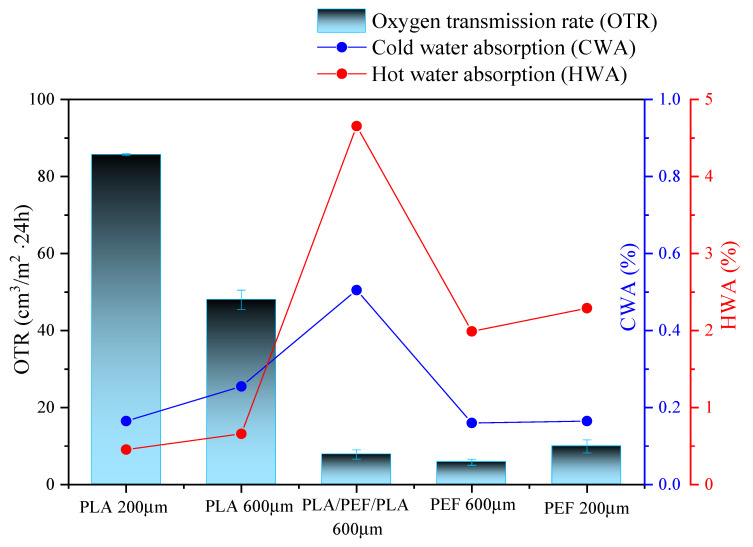
OTR and cold and hot water sorption measurements for the series of polyester foils.

The uptake of water and moisture by polymer materials is influenced by various mechanisms [58,59]: (1) the diffusion of water molecules within micro-gaps or free volume among polymer chains; (2) the capillary movement of water molecules into spaces between two phases due to imperfect interfacial bonding between phases, mainly when they exhibit different polarities and are immiscible; (3) more recently, solubility, particularly in amorphous polymers with low to moderate hydrophilicity [60]. The results of cold water sorption (CWS) and hot water sorption (HWS) are shown in Figure 9. For each sample, three measurements were performed, and the presented results are the arithmetic mean. PLA and PEF foils, regardless of thickness, have very similar absorbency in cold water. However, PEF has a much higher absorbency in boiling water than PLA. The three-layer PLA/PEF/PLA foil showed the highest sorption degree in both cold and boiling water. This effect may be caused by insufficient adhesion between the pressed foils from different materials, as a result of which water could penetrate free spaces between the foils, which could not be removed by drying after testing by the procedure, and which significantly influenced the final result.

In general, due to the presence of polar oxygen linkages, PLA is inherently a hydrophilic polymer. However, the presence of methyl side groups imparts a hydrophobic character to PLA as well [61]. This inherent hydrophilicity contributes to its moderate degradation in response to surrounding moisture and temperature. Previous research indicates that PLA exhibits moderate stability in water at mesophilic temperatures (15–40 °C), with an absorption rate of approximately 0.7 to 1 percent over 30 days [62] (slightly higher with longer immersion periods [63]). In the case of PEF, it was proven that it exhibited a significantly reduced water diffusion coefficient of ~5 times compared to PET at 35 °C [32]. The decrease in the diffusion coefficient of PEF compared to PET stems from the diminished segmental mobility caused by the asymmetrical furan ring present in PEF, as opposed to the symmetrical phenyl ring found in PET [29]. These observations may confirm that the foils based on single-layer foils PLA and PEF, regardless of the thickness, but also on the multilayer system, might be an alternative to PET used in the beverage-contained market and allow the utilization of biobased polyesters in diverse packaging applications.

The oxygen transmission rate (OTR) is the foremost important parameter characterizing the foils and packaging, determining the shelf life of a product [64]. The OTR measurement described by the ASTM D-3985 standard [65] is widely used in industry since the knowledge of the gas permeability properties of packaging materials is crucial for successful packaging design for many products [66]. Gas transmission is affected by the solubility of that permeated in the foil, the diffusion rate through the foil, the thickness of the foil, and the temperature and specific partial pressure difference [67].

The results of the OTR measurements are presented in Figure 9. Presented data [68] show that the permeability of PLA with ca. 200 µm foil thickness is approximately 85 cm^3^/m^2^·24 h. The influence of foil thickness on the improvement of the barrier against oxygen was observed: for PLA600, it was an improvement of 45%. Moreover, according to the literature [61], PEF has been found to exhibit much better barrier properties than PET, over 6 times better concerning O_2_ and 3 times better in relation to CO_2_ [31]. However, based on the obtained results, it can be concluded that the barrier properties of PEF are almost 10 times better than PLA at the same film thickness. The great difference in the OTR value is related to the presence of aromatic rings in furan-based polymers. In turn, the PLA/PEF/PLA multilayer system seems very promising from the point of view of application in the packaging industry. In this case, the effectiveness of the introduction of the PEF as an internal layer as an additional barrier was confirmed, and thus, the system based on 2/3 PLA exhibits barrier properties at the PEF level, and the price of such a system would be significantly lower.

From a technological point of view, i.e., forming products using methods operating in the temperature range between the softening and plastic flow, such as thermoforming or injection blow molding, using a system of two amorphous films is a justified choice. However, considering the conclusions presented by Huang et al. [28], verifying the possibility of using PLA varieties with increased crystallinity would also be essential. While this procedure may prove to be technologically problematic due to material shrinkage narrowing of the temperature processing window and decreasing transparency of final products, the beneficial effects of the increased barrier properties of the PLA layer could allow for limiting the thickness of the outer PLA layers.

### 2.5. Surface Properties

**Figure 10:**
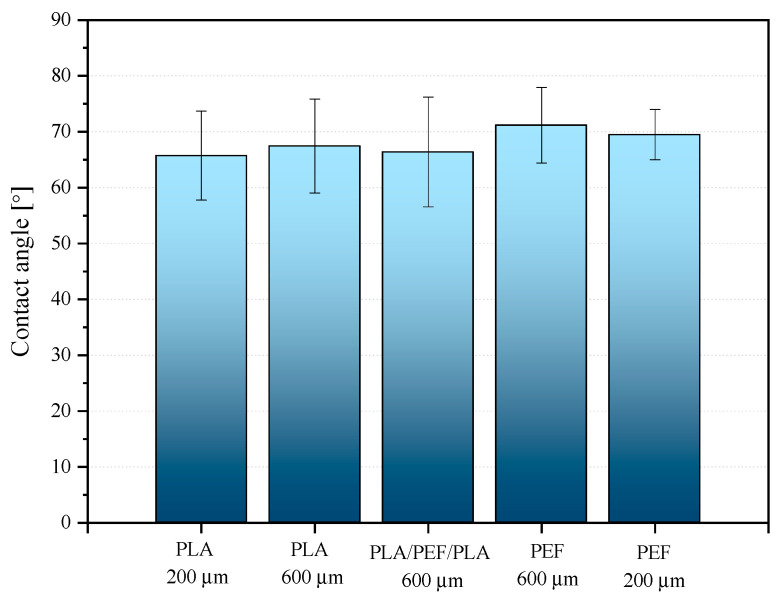
Contact angle results for polyester foils.

The average values of the contact angle measurements are presented in Figure 10. The obtained results are consistent with the literature data [69,70,71]. PEF is more hydrophobic than PLA [69,71]. It should be emphasized that in both cases, the thickness of the measured foil influenced the recorded contact angle value. Both PLA and PEF foils with greater thickness (600 μm) had slightly larger contact angles. Taking into account the amorphous nature of both biobased polyesters and the results of thermal tests, these changes should be related to the change in the surface structure of the formed products resulting from the greater thermal inertia of the thicker foil, which translates into a more uniform surface structure of the product during forming. The contact angle results obtained for the PLA/PEF/PLA laminate indicate well-selected process conditions for this product type. Obtaining a measured value identical to that of 600 μm PLA film suggests that despite the different plastic flow temperatures of both materials and softening temperatures [50], it is possible to form laminates in post-processing using the calendering or high-pressure compression molding method at a forming temperature (Tf) not exceeding Tm without the risk of creating a two-phase blended bulk in the external layer of the thin-walled product.

### 2.6. Preliminary Study of Biofilm Formation on Surface of Polymer Foils

Biofilm formation on the polyester foils’ surface was assessed using Richard’s method, where the following classification of results was adopted:

- (−)—strain not forming the biofilm (corresponded to the lack of cells);
- (+)—strain weakly forming the biofilm (corresponded to 10^3^–10^4^ CFU/mL);
- (++)—strain strongly forming the biofilm (corresponded to 10^5^–10^6^ CFU/mL);
- (+++)—strain strongly forming the biofilm (corresponded to 10^7^–10^8^ CFU/mL).

Pathogenic bacteria like *Escherichia coli, Salmonella enterica,* and *Pseudomonas aeruginosa*, as well as toxigenic bacteria such as *Staphylococcus aureus* and *Bacillus cereus*, are responsible for producing biofilms that pose a threat to the safety of food products [72]. Biofilms contribute to the persistence of these bacteria in food processing environments and the potential (re)contamination of processed foods [73]. When the contamination of food products happens, recalls are necessary [74]. Based on the research conducted, differences in biofilm estimation were observed in the qualitative method, and the results are presented in Table 1. Aliphatic polyesters, like PLA, exhibit not only inherent hydrophilicity, as mentioned above, but are also found to be degradable in model aqueous media or animal bodies [75]. Several studies [74,75,76] identified them as biocompatible and biodegradable polymers that can intentionally undergo chain scissions in the body [76]. It also makes them more susceptible than, for instance, aromatic polyesters, to various bacteria and fungi [74]. Comparing PLA and PEF foils, one can see that PLA (both thicknesses) has a greater affinity towards *S. aureus* strains, which might result from the fact that the ester linkages in PLA are more vulnerable to both chemical hydrolysis and enzymatic cleavage than in PEF [77]. Microorganisms lack transporters to directly absorb plastic polymers into their cells because they are too large and insoluble in water. PEF basically turned out to be a material on which biofilms, both *S. aureus* and *E. coli* strains, are poorly formed. However, this second strain generally formed poorly in all samples. It confirms that PLA is the material with more affinity towards different microorganisms, which, as we explained above, may be due to its greater susceptibility to both chemical and enzymatic degradation. Nonetheless, the multilayer system of PLA/PEF/PLA was the system on which the biofilm formed most effectively. However, herein, perhaps microorganisms could penetrate the spaces between the foils, or the surface of this system was rougher so that the biofilm could have been formed with greater efficiency. Therefore, taking into account the safety of food products and the possibility of obtaining bio-friendly packaging material, it seems justified to choose a multilayer system, which should be subjected to a further analysis, perhaps in terms of the processing or modification of one of the layers of the system exposed to external conditions.

## 3. Experimental Section

### 3.1. Materials

Two types of polyesters were used in this work: PLA and PEF. PLA 4043D (NatureWorks LLC, Minnetonnka, USA) was purchased in the form of ready-made granules and, according to the manufacturer’s data, it is characterized by the following parameters: d = 1.24 g/cm^3^, MFR = 6 g/10 min, T_m_ = 145–160 °C, T_g_ = 55–60 °C, σ_b_ = 53 Mpa, and a D-enantiomer content of 4.5–5% [78] and a molecular weight around 110.000 g/mol [79]. PEF was synthesized from dimethyl 2,5-furanodicarboxylate (DMFDC, 99%, Henan Coreychem Co., Ltd., Zhengzhou, China) and 1,2-etanediol (ED, Sigma-Aldrich, St. Louis, MO, USA) via a two-step polycondensation procedure. In the first step, the transesterification of the diester (DMFDC) by ED in the presence of the first portion of the catalyst (tetrabuthyl orthotitanate, Ti(OBu)_4_, (Sigma-Aldrich)) and the thermal stabilizer, Irganox 1010 (Ciba-Geigy, Basel, Switzerland), was carried out. Secondly, the polycondensation step was performed in the presence of the second portion of the catalyst (also Ti(OBu)_4_). The synthesis was performed in a 1 dm^3^ high-pressure reactor (Autoclave Engineers, Erie, PA, USA) equipped with a condenser, cold trap for collecting the by-product, and vacuum pump. The molar ratio of the diester and glycol was 1:2. During the transesterification, methanol was distilled and collected as the first by-product. The end of the first step was estimated by the amount of effluent by-product. When 90% of the stoichiometric amount of methanol was ceased, the process was completed. Subsequently, one increased the temperature up to the final value (240 °C). A vacuum was applied to remove excess ED and lower the final pressure to 25 Pa. The progress of polycondensation was monitored by the observation of stirring torque change, which was used to evaluate the melt viscosity of the product. The end of the polycondensation process was signalized by the proper value of melt viscosity of the reaction mixture that was adequate relative to the value of melt viscosity of a high molecular mass of the polymer material. After the polycondensation process, the material was extruded from the reactor into the water bath using compressed nitrogen. Finally, the material was granulated, dried, and subjected to further processing.

### 3.2. Sample Preparation

#### 3.2.1. Testing Sample Preparation

The testing samples (dumbbell-shape sample, type A3 according to EN ISO 527-2:2012 [80]) were injection-molded using Boy 15 (Dr Boy GmbH&Co., Neustadt, Germany). Before the melt processing, PLA and PEF granules were dried in a vacuum dryer at 60 °C for 24 h. The following parameters were used for injection molding of PLA—mold temperature: 30 °C, injection temperature: 180 °C, injection pressure: 55 MPa, holding down pressure: 28 MPa, holding down time: 8 s, and cooling time: 20 s, while for PEF, they were mold temperature: 30 °C, injection temperature: 200 °C, injection pressure: 95 MPa, holding down pressure: 29 MPa, holding down time: 8 s, and cooling time: 5 s.

#### 3.2.2. Preparation of Single- and Multilayer Foils

A Colin P200E (Dr. COLLIN GmbH, Ebersberg, Germany) hydraulic press was used to prepare thin foils. PLA was compression-molded into foils with a thickness of 200 μm and 600 μm at a temperature of 210 °C, under a pressure of 5 bar for 2 min and 10 bar for another 2 min. PEF foils, also with thicknesses of 200 μm and 600 μm, were pressed at a temperature of 205 °C under a pressure of 5 bar for 2 min and 10 bar for another 1 min. The three-layer PLA/PEF/PLA laminates were pressed at a temperature of 150 °C and a pressure of 10 bar for a minute. Before compression molding, the samples were dried in a vacuum dryer at 70 °C. The thickness of the polyester foils was measured with a micrometer, no. 293-531, from Mitutoyo.

### 3.3. Characterization Methods

UV-Vis spectra were carried out on a UV-Vis spectrometer (UV-1800, Shimadzu, Kyoto, Japan). Polyester foils were scanned in the 200–800 nm range with a 1 nm interval.

The microscopic examination and measurement of the foils’ thickness were performed using a 4K VHX-7000 digital microscope (Keyence, Osaka, Japan).

Differential scanning calorimetry (DSC) was performed using DSC 204 F1 Phoenix (Netzsch, Selb, Germany). The 10 ± 0.5 mg of each sample was encapsulated in aluminum pans and analyzed in a temperature range from 0 °C to 200 °C for PLA and from −50 °C up to 300 °C for PEF with a 10 °C/min heating/cooling rate. Measurements were carried out under nitrogen flow (20 mL/min) in heating/cooling/heating cycles. The phase transition temperatures, i.e., glass transition, cold crystallization (if visible), and melting, were taken from the second heating run. The crystallization (T_c_), cold crystallization (T_cc_), and melting (T_m_) temperatures were determined from the maximum of the exothermic and endothermic peaks, respectively. Moreover, the degree of crystallinity (*X_c_*) was evaluated according to the following equation:

(1) $$X_{c}=\frac{∆H_{m}-∆H_{cc}}{∆H_{m}^{0}}100\%$$

where Δ*H_m_* and Δ*H_cc_* are the enthalpy of melting and cold crystallization (if observed), respectively; $∆H_{m}^{0}\;$is the enthalpy of melting of a 100% crystalline sample depending on the type of material for PLA at 93 J/g [81] and PEF at 137 J/g [82].

The tensile properties for injection-molded samples and the foils were measured according to PN-EN ISO 527 [80] and PN-EN ISO 178 [83] using an Autograph AG-X plus (Shimadzu, Kyoto, Japan) tensile testing machine equipped with a 1 kN Shimadzu load cell, a contact optical long-travel extensometer, and TRAPEZIUM X computer software version 1.4.5, operated at a constant crosshead speed of 5 mm/min. Measurements were performed at room temperature on the dumbbell samples (type A3) with a grip distance of 20 mm. Young’s modulus (E), strength at break (σ_b_), and elongation at break (ε_b_) for the injection-molded samples and the prepared thin foils were determined. Five measurements were conducted for each sample, and the results were averaged to obtain a mean value.

The limiting viscosity number (LVN) of PLA and PEF was measured at 30 °C in the mixture of phenol/1,1,2,2-tetrachloroethane (60/40 by weight), while the concentration of the polymer solution was 0.5 g/dL. The measurement was carried out using an Ubbelohde capillary viscometer (type Ic, K = 0.03294).

The water sorption test followed the procedures outlined in ASTM D570, and was carried out in cold and boiling water. Dumbbell-shaped samples were dried to a constant mass at 50 °C within 24 h, cooled to room temperature, and weighed. For the assessment of cold water sorption, the specimens were immersed in distilled water at 23 °C for 24 h. Boiling water immersion was conducted for 30 min, after which the specimens were allowed to cool to room temperature in distilled water. Any surface water was removed with filter paper before weighing the samples. Each reported value represents the average of five test specimens.

The oxygen transmission rate (OTR) was assessed using an Ox-Tran 2/10 instrument (Mocon, Mocon, Minneapolis, MN, USA), with 5 cm² samples of polymer films tested according to ASTM D3985-05 [84] and a standard. Before testing, all polyester foils were conditioned for 3 h in the test chamber of the apparatus with test parameters (23 °C and 0% relative humidity—RH).

Surface wettability was studied through static water contact angle measurements using an Ossila L2004 contact angle goniometer (Ossila Ltd., Sheffield, UK) equipped with a camera and Ossila Contact Angle v. 3.0.9.1 software. Ten contact angle measurements were taken in random positions, putting drops of ~1 µL distilled water onto the samples’ surface with a syringe. The average values were calculated and reported.

In the microbiological study, two reference strains, *Staphylococcus aureus* ATCC 25923 and *Escherichia coli* ATCC 25922, were used. The Columbia agar medium with 5% sheep blood (bioMérieux, Warsaw, Poland) was used for breeding bacteria. Incubation was performed at 37 °C for 24 h under aerobic conditions. The study of the formation of bacterial biofilms on the surface of thin polyester foils cut to 1:1 cm was estimated using Richards’ qualitative method following the procedure described in [85].

## 4. Conclusions

In this work, a series of polyester foils differing in thickness (200 and 600 μm) and a PLA/PEF/PLA multilayer system, defined as a laminate, based on biobased thermoplastic polyesters were prepared. Commercially available PLA (PLA Ingeo^TM^ 4043D, NatureWorks LLC, Minnetonnka, MN, USA) and PEF synthesized by melt polycondensation were used to prepare the analyzed foils. The obtained materials were examined for their basic physicochemical properties, characteristic phase transition temperatures, and mechanical and barrier properties. In addition, the susceptibility of foils to biofilm formation on their surface was studied.

In the present study, an attempt was made to determine whether it would be more advantageous both economically and environmentally but also technologically to use a single-layer foil of a certain thickness or a three-layer one. Based on the obtained results, it was determined that the multilayer system exhibited the most promising mechanical properties: the value of the elastic modulus was significantly higher compared to single-layer foils at the same thickness. On the other hand, in the case of monolayer foils, higher-strength parameters were observed for PLA foils, which could be due to higher LVN values. On the other hand, regardless of thickness, PLA and PEF films show very similar absorption values in cold water. In contrast, in boiling water, PEF films showed a significantly higher sorption degree than PLA films. The PLA/PEF/PLA three-layer system showed the highest absorbency in cold and boiling water, which could be due to the penetration of water molecules into the spaces between the layers with insufficient adhesion.

Moreover, PLA foils were slightly more transparent than PEF foils, while the multilayer system was the least transparent, even compared to single-layer foils of comparable thicknesses. PEF foils were shown to be superior in terms of barriers to oxygen molecules than PLA series (at the same sample thickness, PEF 200 μm foil is practically 10 times less permeable to oxygen than PLA 200 μm foil). Additionally, the effect of foil thickness on the OTR value was demonstrated: thus, PLA at 600 μm shows an OTR value almost half that of PLA at 200 μm, while for PEF foils, this difference is about 30%. In contrast, the multilayer system, consisting of two PLA external layers and one internal PEF core, showed an OTR value only slightly higher than the PEF film. This is a promising result, confirming the sensibility of the research topic undertaken in this work on the application of biobased thermoplastic polyesters in the packaging industry. The prepared PLA/PEF/PLA system shows outstanding strength characteristics and barriers to oxygen molecules comparable to PEF films, while the final price of such a system would be 60% lower. In addition, PEF was found to be a material that exhibited a limited formation of bacterial biofilms, i.e., strains of *S. aureus* and *E. coli*. However, the latter strain generally showed poor formation across all samples.

## 5. Patents

The patent application P.450381 titled “Aromatic-aliphatic polyester laminates with improved barrier properties intended for packaging applications (*Laminaty poliestrowe alifatyczno-aromatyczne o polepszonej barierowości do zastosowań w opakowalnictwie*)” resulted from the work reported in this manuscript.

## Figures and Tables

**Table 1:** Classification of biofilm formation for individual strains.

Material	Strain Used in Biofilm Analysis	
	*S. aureus*	*E. coli*
PLA, 200 μm	++	+
PLA, 600 μm	++	+
PLA/PEF/PLA, 600 μm	+++	+
PEF, 600 μm	+	+
PEF, 200 μm	+	+

## Data Availability

The data presented in this study are available on request from the corresponding author.

## Supplementary Materials

The following supporting information can be downloaded at: https://www.mdpi.com/article/10.3390/molecules30010178/s1, Table S1: Characteristic phase transition temperatures, corresponding enthalpies and changes in heat capacity, and the values of degrees of crystallinity.
