# Supplementary material for: Sustainable and Eco-Friendly Single- and Multilayer Polyester Foils (Laminates) from Polylactide and Poly(Ethylene 2,5-Furandicarboxylate)
Source: Molecules. 2025 Jan 4;30(1):178. doi: 10.3390/molecules30010178 (PMC11721795; doi:10.3390/molecules30010178)
Supplement: Supplementary file 1 [file molecules-30-00178-s001.zip › molecules-3374114-supplementary.pdf]

Supplementary materials

Table 1_SM. Characteristic phase transition temperatures, corresponding enthalpies and changes in heat capacity, and the values of degrees of crystallinity

<table>
<tr><th rowspan="2">Material</th><th colspan="11">1st heating</th><th colspan="2">Cooling</th><th colspan="9">2nd heating</th></tr>
<tr><th colspan="2">T_g [ºC]</th><th colspan="2">ΔC_{p1} [J/g·ºC]</th><th colspan="2">T_{cc1} [ºC]</th><th colspan="2">ΔH_{cc1} [J/g]</th><th>T_{m1} [ºC]</th><th>ΔH_{m1} [J/g]</th><th>X_{c1} [%]</th><th>T_g [ºC]</th><th>ΔC_p [J/g ºC]</th><th colspan="2">T_{g2} [ºC]</th><th colspan="2">ΔC_{p2} [J/g·ºC]</th><th>T_{cc2} [ºC]</th><th>ΔH_{cc2} [J/g]</th><th>T_{m2} [ºC]</th><th>ΔH_{m2} [J/g]</th><th>X_{c2} [%]</th></tr>
<tr><td>PLA granule</td><td colspan="2">63.1</td><td colspan="2">0.505</td><td colspan="2">-</td><td colspan="2">-</td><td>151.8</td><td>41.8</td><td>44.9</td><td>55.2</td><td>0.324</td><td colspan="2">59.8</td><td colspan="2">0.704</td><td>-</td><td>-</td><td>152.6</td><td>1.0</td><td>1,1</td></tr>
<tr><td>PLA foil, 200µm</td><td colspan="2">57.1</td><td colspan="2">0.489</td><td colspan="2">115.0</td><td colspan="2">25.5</td><td>150.0</td><td>27.7</td><td>2.4</td><td>55.1</td><td>0.384</td><td colspan="2">58.7</td><td colspan="2">0.552</td><td>119.6</td><td>24.1</td><td>151.7</td><td>26.4</td><td>2.5</td></tr>
<tr><td>PLA foil, 600µm</td><td colspan="2">55.6</td><td colspan="2">0.506</td><td colspan="2">118.1</td><td colspan="2">23.6</td><td>150.4</td><td>25.2</td><td>1.7</td><td>55.4</td><td>0.401</td><td colspan="2">58.1</td><td colspan="2">0.600</td><td>118.9</td><td>23.3</td><td>150.7</td><td>24.7</td><td>1.5</td></tr>
<tr><td>PEF granule</td><td colspan="2">66.6</td><td colspan="2">0.463</td><td colspan="2">-</td><td colspan="2">-</td><td>-</td><td>-</td><td>-</td><td>72.7</td><td>0.408</td><td colspan="2">76.5</td><td colspan="2">0.399</td><td>-</td><td>-</td><td>-</td><td>-</td><td>-</td></tr>
<tr><td>PEF foil, 200µm</td><td colspan="2">67.2</td><td colspan="2">0.400</td><td colspan="2">-</td><td colspan="2">-</td><td>-</td><td>-</td><td>-</td><td>72.6</td><td>0.302</td><td colspan="2">75.9</td><td colspan="2">0.298</td><td>-</td><td>-</td><td>-</td><td>-</td><td>-</td></tr>
<tr><td>PEF foil, 600µm</td><td colspan="2">66.0</td><td colspan="2">0.524</td><td colspan="2">-</td><td colspan="2">-</td><td>-</td><td>-</td><td>-</td><td>72.4</td><td>0.410</td><td colspan="2">74.8</td><td colspan="2">0.447</td><td>-</td><td></td><td>-</td><td></td><td>-</td></tr>
<tr><td rowspan="2">PLA/PEF/PLA laminate, 600µm</td><td>PLA</td><td>PEF</td><td>PLA</td><td>PEF</td><td colspan="7">PLA</td><td colspan="2">PLA</td><td>PLA</td><td>PEF</td><td>PLA</td><td>PEF</td><td colspan="5">-</td></tr>
<tr><td>53.3</td><td>72.3</td><td>0.169</td><td>0.040</td><td colspan="2">110.3</td><td colspan="2">16.9</td><td>150.1</td><td>18.9</td><td>2.2</td><td>50.2</td><td>0.282</td><td>54.7</td><td>79.2</td><td>0.551</td><td>0.040</td><td>-</td><td>-</td><td>-</td><td>-</td><td>-</td></tr>
</table>

Where: T_{gx} - glass transition temperature, ΔC_{px} - change of heat capacity, T_{ccx} – cold crystallization temperature, ΔH_{ccx}– enthalpy of cold crystallization, T_{mx} – melting temperature, ΔH_{mx}– enthalpy of melting, X_{cx} – degree of crystallinity, x - subscripts of 1 and 2 apply to individual heating cycles
